# Cornual Pregnancy: A Rare Occurrence Managed Laparoscopically

**DOI:** 10.7759/cureus.107977

**Published:** 2026-04-29

**Authors:** Dur-e- Shahwar, Kaneez Fatima

**Affiliations:** 1 Obstetrics and Gynecology, Patel Hospital, Karachi, PAK

**Keywords:** corneal resection, cornual ectopic pregnancy, ectopic pregnancy, minimal invasive surgery, unruptured ectopic pregnancy

## Abstract

Cornual ectopic pregnancy, defined as implantation in the interstitial (cornual) portion of the fallopian tube, is a rare and complex form of ectopic pregnancy. Due to its infrequent occurrence, there is no universally accepted standard protocol or uniform management strategy. Similar to other ectopic pregnancies, both medical and surgical treatment options are available. Although ultrasound remains the gold standard imaging modality for diagnosing ectopic pregnancy, clinical evaluation continues to play a crucial role in early identification.

Here, we describe a case of a 38-year-old Asian woman who presented at 8+ weeks of gestational amenorrhea with acute onset of severe lower abdominal pain. Based on serum beta-human chorionic gonadotropin (β-hCG) levels and gestational sac size, a laparoscopic approach was undertaken. A cornual pregnancy was diagnosed intraoperatively and successfully managed laparoscopically with minimal blood loss.

## Introduction

Usually, a pregnancy implants within the uterine cavity; however, when an embryo implants outside the uterine cavity, it is termed an ectopic pregnancy [1,2]. Ectopic pregnancies are generally diagnosed early, typically in the first trimester, but they may be overlooked until complications occur, such as rupture [1,2]. This condition inherently predisposes patients to a higher risk of hemorrhage [2].

Approximately 98% of ectopic pregnancies occur in the fallopian tube, with about 80% of these located in the ampullary region. In contrast, only around 2% of ectopic pregnancies occur in the cornual (interstitial) region of the fallopian tube, which is the portion that traverses the muscular layer of the uterus [3,4]. This region is highly vascularized, increasing the risk of significant hemorrhage.

Management is often challenging due to the rarity of these cases. Available options include expectant, medical, and surgical management. The choice of approach depends on multiple factors, including the patient’s clinical status, serum beta-human chorionic gonadotropin (β-hCG) level, gestational sac size, and other considerations. Regarding surgical management, laparotomy was previously widely performed; however, with advances in and increasing expertise in minimally invasive techniques, laparoscopy has become the preferred approach worldwide for the management of ectopic pregnancies [3]. Although laparoscopy offers advantages such as reduced postoperative pain, minimal blood loss, shorter hospital stay, faster recovery, improved cosmetic outcomes, and a lower risk of wound-related complications compared with open surgery, it requires significant surgical expertise. Laparoscopic resection of the cornual region with effective hemostasis, achieved through the injection of diluted vasopressin, is an optimal approach for controlling bleeding while facilitating rapid recovery.

## Case presentation

A 38-year-old Asian woman, gravida 5, para 0+4, presented to the emergency department at 8+ weeks of gestational amenorrhea with complaints of mild lower abdominal pain and spotting for the past week. On presentation, she appeared anxious and uncomfortable. She was of average height and build, lying in bed. Her blood pressure was 100/72 mmHg, pulse rate 110 beats/min, temperature 98.6°F, and respiratory rate 20 breaths/min.

On chest auscultation, bilateral equal air entry was noted. Abdominal examination revealed a soft abdomen that was non-tender on superficial palpation; however, mild rebound tenderness was elicited in the suprapubic region on deep palpation. On per speculum examination, a mild brown-colored, thick discharge was observed from the cervical os. On bimanual vaginal examination, mild tenderness was noted.

She had no known medical comorbidities apart from occasional seasonal flu and no known allergies. Her obstetric history was significant for three early pregnancy losses and one mid-trimester loss at 21 completed weeks of gestation.

**Figure 1:**
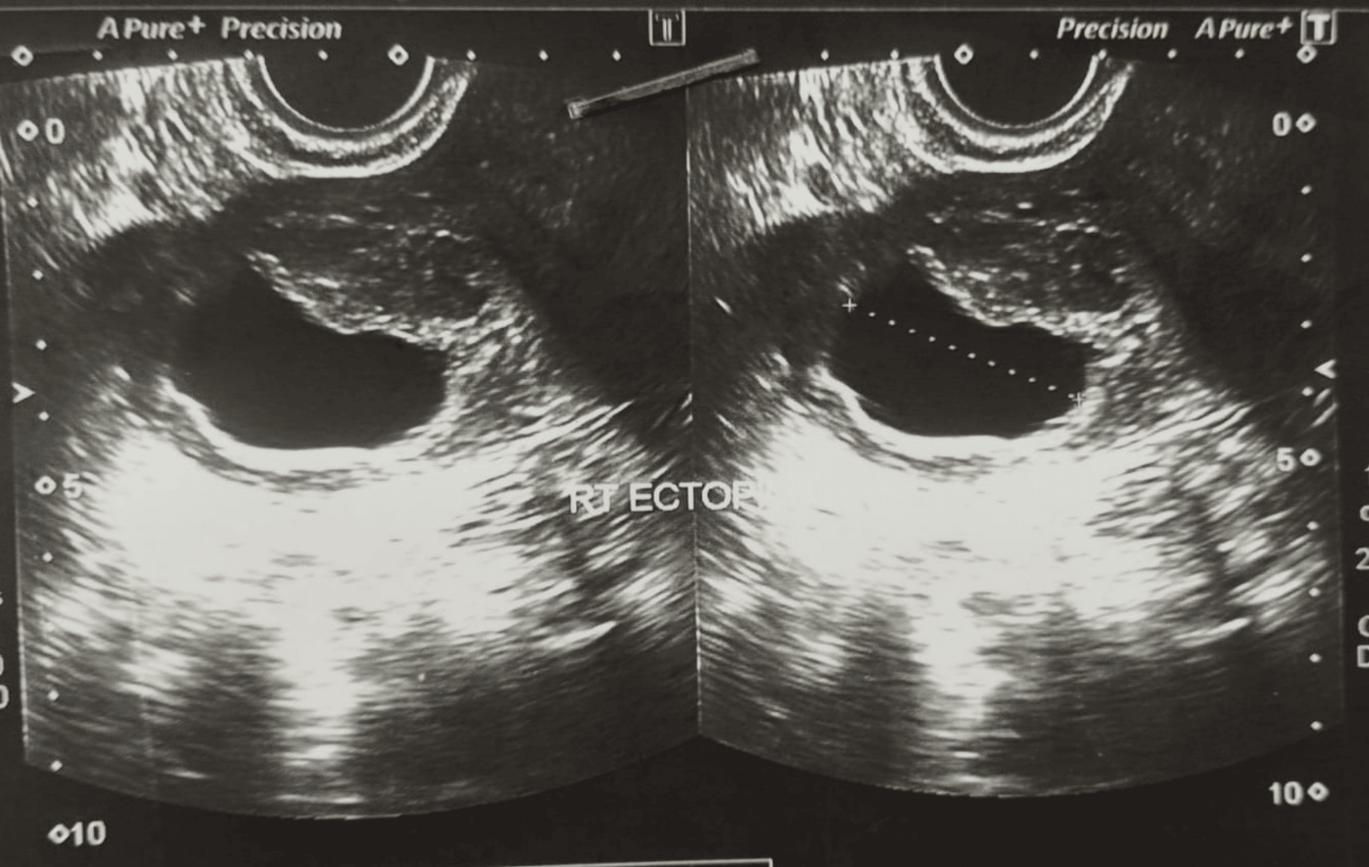
Ultrasound image demonstrating a right-sided ectopic pregnancy

On initial workup, her serum β-hCG level was 6,065 mIU/mL. Transvaginal ultrasonography revealed an ectopic pregnancy corresponding to 8+5 weeks of gestation in the right adnexa, with a crown-rump length of 1.1 cm and a gestational sac measuring 3.8 × 3.6 cm (Figure 1).

The patient developed sudden, severe lower abdominal pain within two hours of presentation to the emergency department. Based on clinical and radiological findings, a decision was made to proceed with diagnostic laparoscopy, with a senior anesthetist and obstetrician involved in the management.

**Figure 2:**
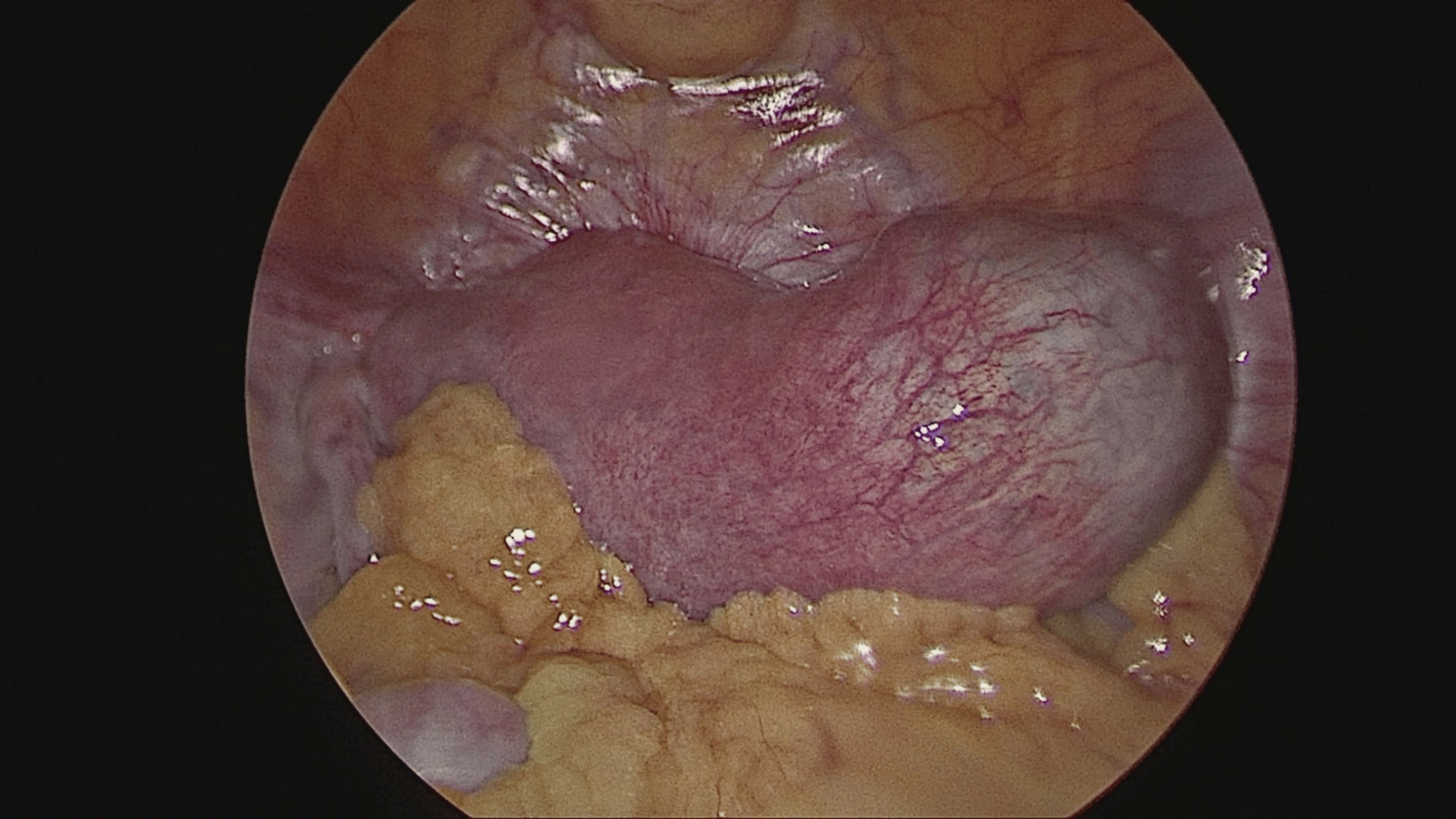
Intraoperative view of a cornual pregnancy, with the ectopic gestation located at the uterine cornua

**Figure 3:**
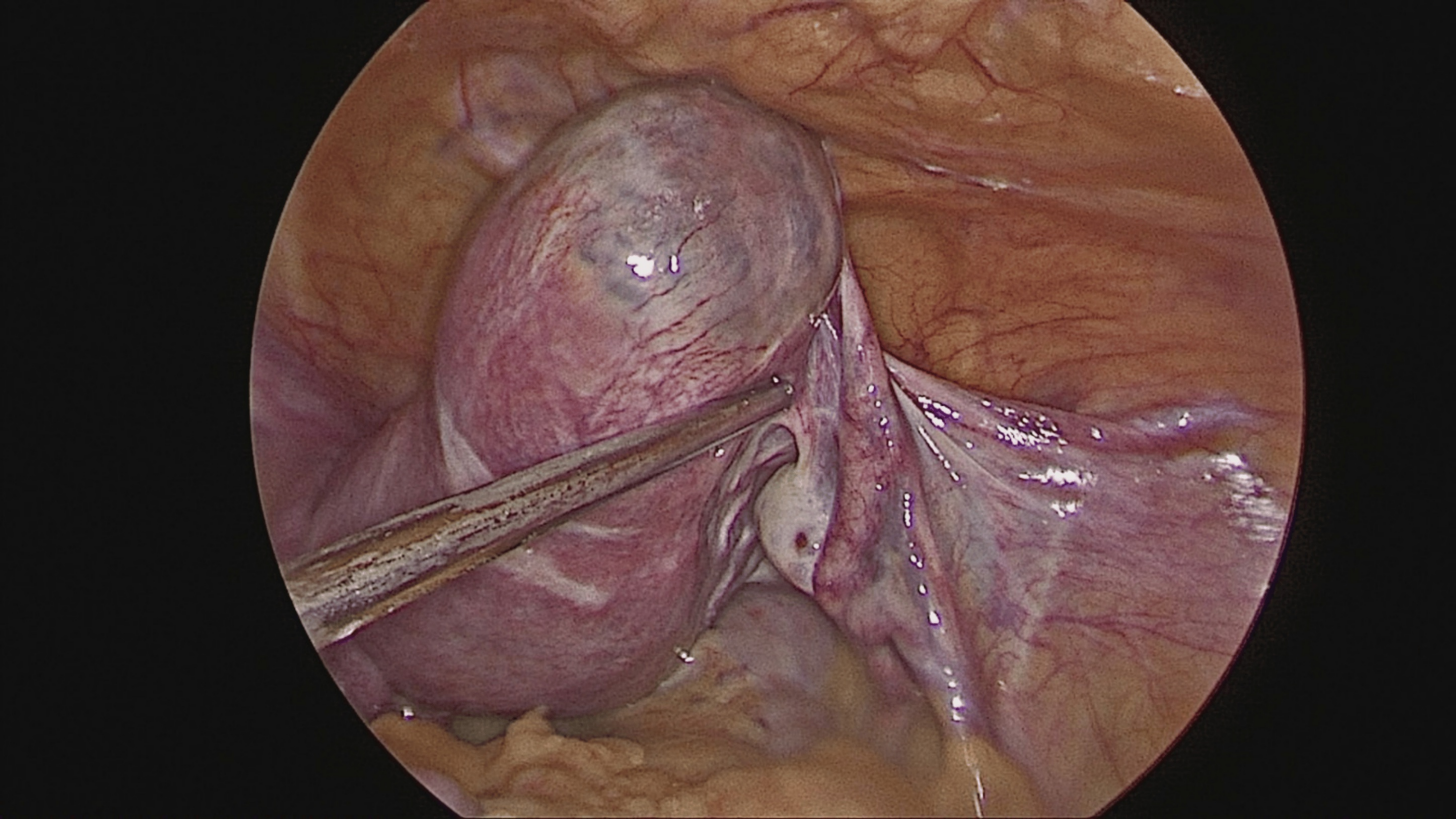
Right-sided cornual pregnancy showing an ectopic gestation located near the right fallopian tube

**Figure 4:**
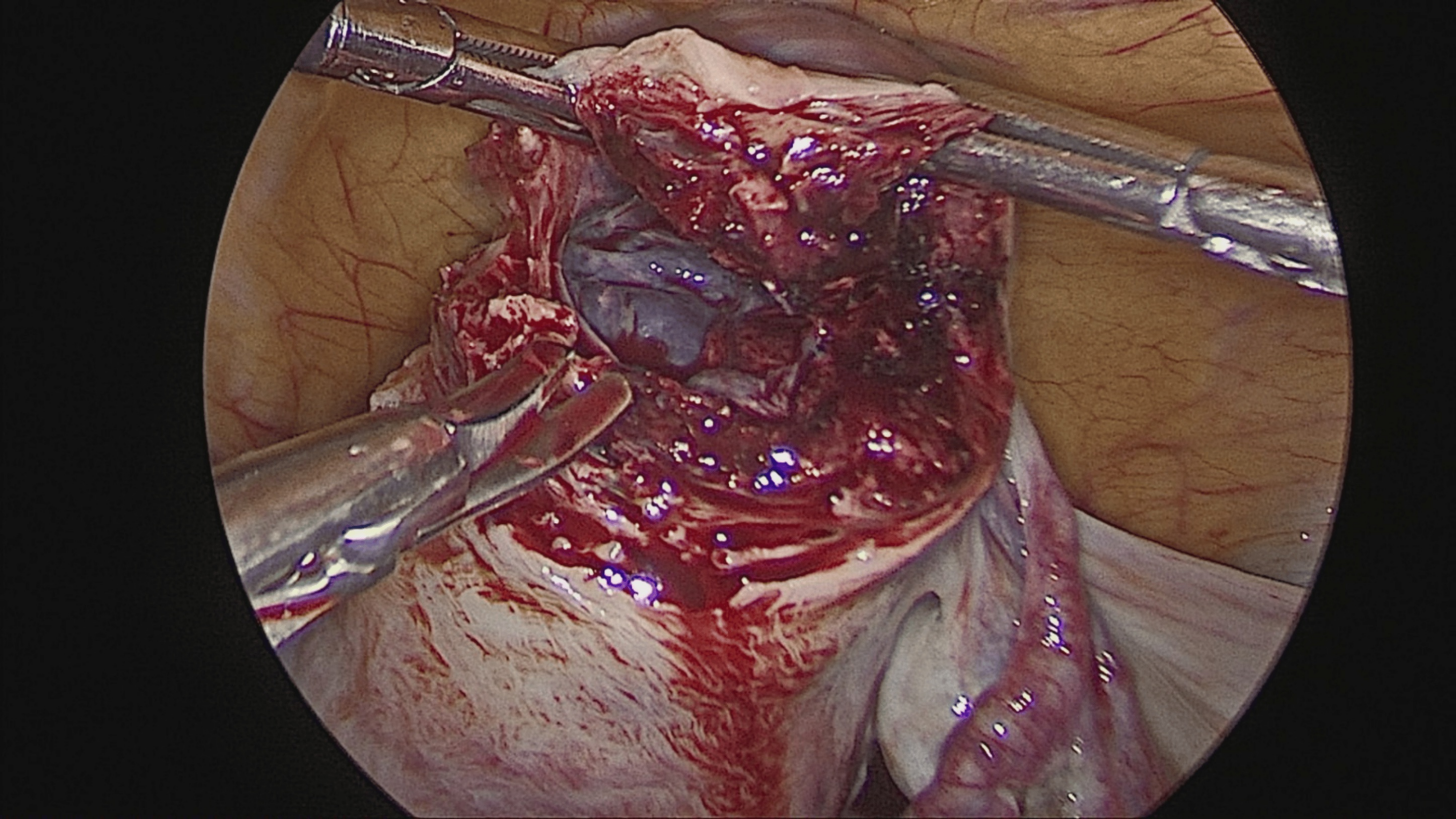
Gestational sac visualized at the uterine cornua during dissection

**Figure 5:**
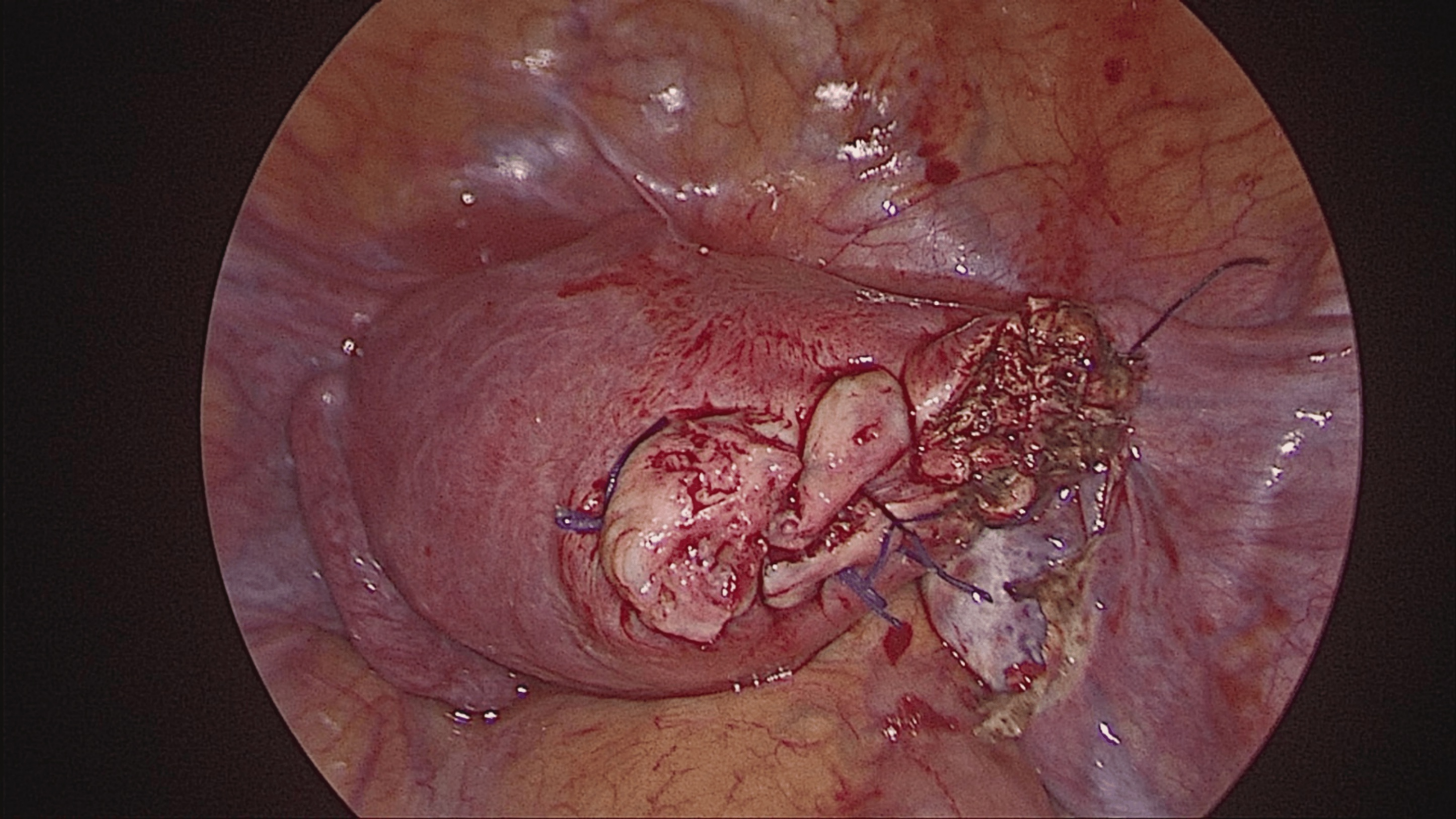
Post-resection view following excision of the cornual pregnancy

After induction of general anesthesia and endotracheal intubation, the patient was cleaned and draped in the Trendelenburg position. A 10 mm supraumbilical primary port was inserted using the direct entry technique, and pneumoperitoneum was established. A right-sided unruptured cornual ectopic pregnancy measuring approximately 3 × 4 cm was identified (Figures 2-3). Diluted vasopressin was injected to aid hemostasis, followed by careful dissection. A 2 × 2 cm gestational sac along with trophoblastic tissue was removed, along with the right fallopian tube, and sent for histopathological examination (Figure 4). Hemostasis was achieved using baseball sutures (Figure 5).

The patient remained hemodynamically and clinically stable in the postoperative period. She was mobilized on the day of surgery and discharged after 18 hours. She resumed her daily activities within three days and attended a routine follow-up visit on the 10th postoperative day. Histopathological examination confirmed the presence of products of conception.

Based on her history, clinical findings, ultrasound report, and β-hCG levels, laparoscopic management had been planned. To our knowledge, this represents a rare form of ectopic pregnancy successfully managed using a minimally invasive approach with careful technique and favorable postoperative recovery.

## Discussion

Ectopic pregnancy refers to implantation of a fertilized ovum outside the uterine cavity and represents an undesirable outcome of conception. It may occur at various sites, with the fallopian tube being the most common location. Other sites include the ovary, cervix, interstitium, cornua, and, rarely, the abdominal cavity. Cornual pregnancy is one of the rare forms of ectopic pregnancy [1,2]. Clinical presentation varies widely, ranging from mild to severe lower abdominal pain and occasionally syncope. In some cases, diagnosis can be challenging [3]. Timely recognition is crucial, as even a short delay may result in significant maternal morbidity and mortality. The classical clinical triad consists of abdominal or pelvic pain, amenorrhea followed by vaginal bleeding, and elevated human chorionic gonadotropin (β-hCG) levels [4].

Predisposing factors include a range of conditions that compromise tubal function or motility, such as inflammatory damage to the fallopian tubes (e.g., salpingitis or *Chlamydia trachomatis* infection), prior tubal ligation, infertility unrelated to tubal pathology, ovulation induction therapies, previous ectopic pregnancy, history of tubal surgery, smoking, in utero exposure to diethylstilbestrol, and advanced maternal age [4].

Cornual ectopic pregnancies are often difficult to diagnose due to their uncommon location and close proximity to the uterine cavity. Consequently, distinguishing cornual ectopic pregnancies from other eccentrically located intrauterine gestations can be challenging [4], potentially leading to delayed or missed diagnosis [5,6].

Unlike other ectopic pregnancies, patients with cornual pregnancies tend to present later, usually in the late first trimester, further complicating early detection. As illustrated in Figure 1, the interstitial (cornual) segment of the fallopian tube traverses the myometrial wall of the uterus. Owing to the rich vascularity of this region and the ability of the myometrium to distend, rupture of a cornual ectopic pregnancy can result in massive hemorrhage, similar to other ectopic sites, but often more severe. Hemorrhagic shock occurs in nearly one-quarter of patients presenting with rupture-related hypovolemia, contributing significantly to maternal morbidity and mortality. The rate of hysterectomy following rupture is reported to be approximately 40%, and in pregnancies progressing beyond 12 weeks, there is an estimated 20% risk of uterine rupture [7,8].

## Conclusions

Here, we present a case of ectopic pregnancy that was diagnosed intraoperatively as a cornual ectopic pregnancy and managed laparoscopically. The procedure involved initial injection of vasopressin, followed by dissection of the cornual ectopic tissue and repair using baseball sutures. The patient had an uneventful postoperative recovery, with the advantages of laparoscopy, including minimal blood loss, a shorter hospital stay, and an early return to routine activities. This case highlights evolving surgical expertise in the laparoscopic management of ectopic pregnancies at uncommon sites, demonstrating its feasibility and safety in complex presentations.
